# Induced Susceptibility of Host Is Associated with an Impaired Antioxidant System Following Infection with *Cryptosporidium parvum* in Se-Deficient Mice

**DOI:** 10.1371/journal.pone.0004628

**Published:** 2009-02-27

**Authors:** Chengmin Wang, Yanyun Wu, Jianhua Qin, Haoxue Sun, Hongxuan He

**Affiliations:** 1 National Research Center For Wildlife Born Diseases, Key Laboratory of Animal Ecology and Conservation Biology, Institute of Zoology, Chinese Academy of Sciences, Beijing, People's Republic of China; 2 Department of Animal Science and Technology, Hebei Agricultural University, Baoding, Hebei, People's Republic of China; Instituto Oswaldo Cruz and FIOCRUZ, Brazil

## Abstract

**Background:**

Susceptibility or resistance to infection with *Cryptosporidium parvum* (*C.parvum*) correlates with Selenium (Se) deficiency in response to infection. Both adult Se-adequate and Se-deficient mouse models of cryptosporidiosis were used to study the cell-mediated immune response during the course of *C. parvum* infection.

**Methodology/Principal Findings:**

Blood samples from mouse models were used for Se status. The concentration of MDA, SOD, GPx and CAT in blood has revealed that lower Se level exist in Se-deficient mice. Mesenteric lymph node (MLN) lymphocytes from both mouse models were proliferated after ex vivo re-stimulation with *C. parvum* sporozoite antigen. The study of the cytokine profiles from the supernatant of proliferated MLN cells revealed that Se-adequate mice produced higher levels of Th1 (IFN-γ and IL-2) and moderate amounts of Th2 (IL-4) cytokines throughout the course of infection. Whereas, MLN cells from Se-deficient mice produced lower levels of IFN-γ, IL-2 and IL-4 cytokines. The counts of total white cell and CD3, CD4, CD8 cell in Se-adequate were higher than that in Se-deficient mice.

**Significance:**

These results suggest that Cell immunity is affected by Se status after infection with *C.parvum* from kinetic changes of different white cells and cytokine. In conclusion, induced susceptibility of host is associated with an impaired antioxidant system following infection with *C.parvum* in C57BL/6 Selenium deficient mice.

## Introduction

*Cryptosporidium parvum* (*C.parvum*) is a coccidian parasite that infects the microvillous region of epithelial cells lining the digestive and respiratory tracts of vertebrates [1], [2]. In immuno-competent hosts, *C. parvum* generally causes a short-term diarrheal illness that resolves spontaneously [3], [4]. However, in immuno-compromised hosts, *C. parvum* may cause a life-threatening, prolonged, choleralike illness.

Generally, Selenium (Se) is a trace element essential to organisms from bacteria to humans. One of its main functions is an anti-oxidant action, involved in protection against damage caused by free radicals and oxidative stress [5], [6]. Se is considered to be an essential component of SeCys in several specific selenoproteins and can promote cell proliferation, a fact of particular importance to the immune response [7]. Se protects membrane lipids against oxidation and results in peroxide destruction, i.e., the reduction of hydrogen peroxide and organic peroxides, reduction of the oxidized glutathione form. The antioxidant properties of Se producing a protective barrier against free radicals play an important role in numerous metabolic and immunological processes associated with the oxidation-reduction reactions [8], [9] which are involved in intracellular digestion of phagocyted bacteria. In mammals, Se exerts its physiological activity in the form of selenoproteins, such as glutathione peroxidase (GPx), superoxide dismutase (SOD), glutathione reductase (GR), thioredoxin reductase (TR), selenoprotein P and selenoprotein W [10], [11].

Se is found in all organs and its deficiency is associated with various metabolic disorders while even its border excess is highly toxic for metabolic processes and immune mediators. A complex antioxidant defense system consists of antioxidant enzymes (GPx, TR, catalase (CAT), GR, SOD family and so on,) metal-binding and redox proteins (transferrin, ceruloplasmin, and thioredoxin (TRx)), and numerous low- molecular-weight antioxidants, including glutathione (GSH), and vitamins C and E [11].

The studies presented here demonstrate the kinetics and comparative analysis of *C. parvum*-specific cell-mediated immune responses to *C. parvum* and Se status before or after infection in Se-deficient and Se-adequate mice models. The purpose of this study was to determine whether Se deficiency improves susceptibility of host to infection of *C.parvum* under the conditions of Selenium deficiency. All of the results in this study indicate that Se deficiency can result in the change of anti-oxidant and immune status of host and increasing susceptibility to *C.parvum*. In conclusion, it is important significance for Se deficient area to prevent the infection of *C.parvum* in nutritional element aspect.

## Results

### Analysis of nutritional selenium depletion

**Figure 1:**
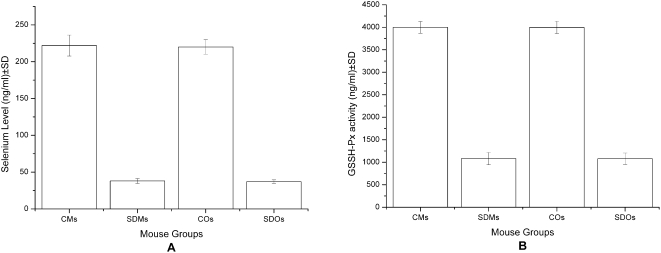
Selenium deficient C57BL/6 mice model by Se depletion of mother Selenium levels (A) and GPx activity (B) from the groups of Control Mothers and Se deficient Mothers. CM represents Control Mothers (N = 20), and SDM represents Se-Deficient Mothers (N = 20). CO represents Control Offspring (N = 20), and SDO represents Se-Deficient Offspring (N = 20). Results are expressed as the mean concentration per group±standard deviation (SD).

Se deficiency was performed by feeding 14 days pregnant female mice with Se deficient chow. Measurements of Se levels and of GPx activity in the group Se deficient Mothers confirmed the deficiency (Figure 1), while Control Mothers and Control Offspring had mean levels of 222 ng and 220 ng Se/ml, 3.998 U and 3.996 U GPx/ml, in Se deficient Mothers and Offspring the values were 38 ng and 37 ng Se/ml, 1.080 U and 1.078 U GPx/ml, respectively. Se-deficient chow administered during mating, pregnancy and lactation led to a reduction of 5.8-and 3.7-times, respectively of Se and GPx in the mothers.

### Determination of antioxidant enzyme activity

**Figure 2:**
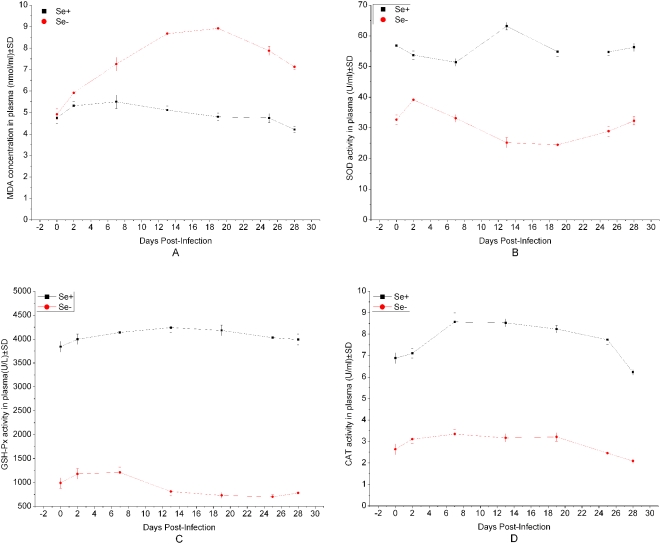
The Kinetics of antioxidant enzymes activity in plasma of Se-deficient and-adequate mice at different days after inoculation with *C. parvum*. The blood of all mice (Se-deficient mice = 20; Se-adequate mice = 20) were collected and then the plasma were separated to measure concentration of MDA, activity of SOD,GSH-Px and CAT at day 0, 2,7,13,19,25 and 28p.i.. A, the concentration of MDA; B, the activity of SOD; C, the activity of GSH-Px; D, the activity of CAT. There were extremely significant difference between the two groups (p<0.01) in activity of SOD, GSH-Px, MDA and CAT at day 0, 2, 7,13,19,25 and 28p.i. However, there were no significant difference (p>0.05) between the two groups in the concentration of MDA at day 0 and 28p.i., extremely significant difference from day 7 to day 28 p.i. (p<0.01). Results are expressed as the mean concentration per group±standard deviation (SD).

After inoculation of *C.parvum*, the total concentration of MDA in the plasma samples of Se-deficient mice was markedly higher than that of Se-adequate mice (p<0.05), especially there was extremely significant difference between two groups (p<0.01) at day 13 and day 19 p.i. (Figure 2A). The activity of SOD in Se-deficient group was lower than that in Se-adequate group (p<0.05), but the difference was extremely significant (p<0.01) at day 13 p.i. (Figure 2B). The activity of GPx in the plasma of mice infection with *C.parvum* initially increased gradually, and then decreased slowly (p<0.05) (Figure 2C). Simultaneously slow decrease of CAT activity was observed. However, the total activity of SOD in Se-deficient group was lower than that of Se-adequate group (p<0.05), especially extremely significant difference (p<0.01) between the two groups at day 13 and day 19 (Figure 2D).

### Oocyst shedding and body weight profiles of Se-deficient and Se-adequate mice

**Figure 3:**
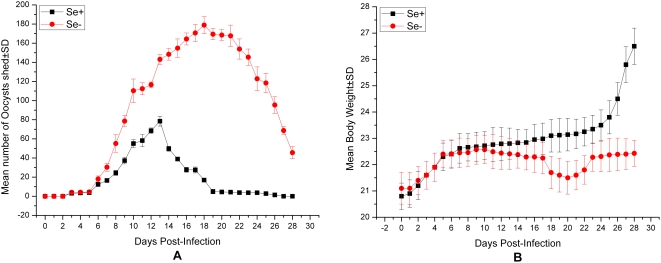
Kinetics of mean oocyst shedding and body weights following the infection of *C. parvum*. Mean oocyst shedding (A) and body weights (B) of *Cryptosporidium parvum*-infected Se-deficient and Se-adequate mice. Se-deficient and Se-adequate mice were orally inoculated with 1×10^6^ or 2.5×10^3^ *C. parvum* oocysts, respectively. The level of oocysts shed in the feces and body weight of each mouse were assessed one times per day. Results are expressed as the mean number of oocysts score or mean body weight per group±standard deviation (SD).

To evaluate the effect of Selenium on Se-deficient and Se-adequate mice models, oocyst shedding level (Figure 3A) and the body weight (Figure 3B) of each mouse were examined every day following infection with *C. parvum*. Significant variations in the patterns of oocyst shedding and weight loss were observed between Se-deficient and Se-adequate mice. The levels of oocyst shedding and mean body weights for Se-deficient and Se-adequate mice were shown in Figure 3.

### Proliferative response to C. parvum

**Figure 4:**
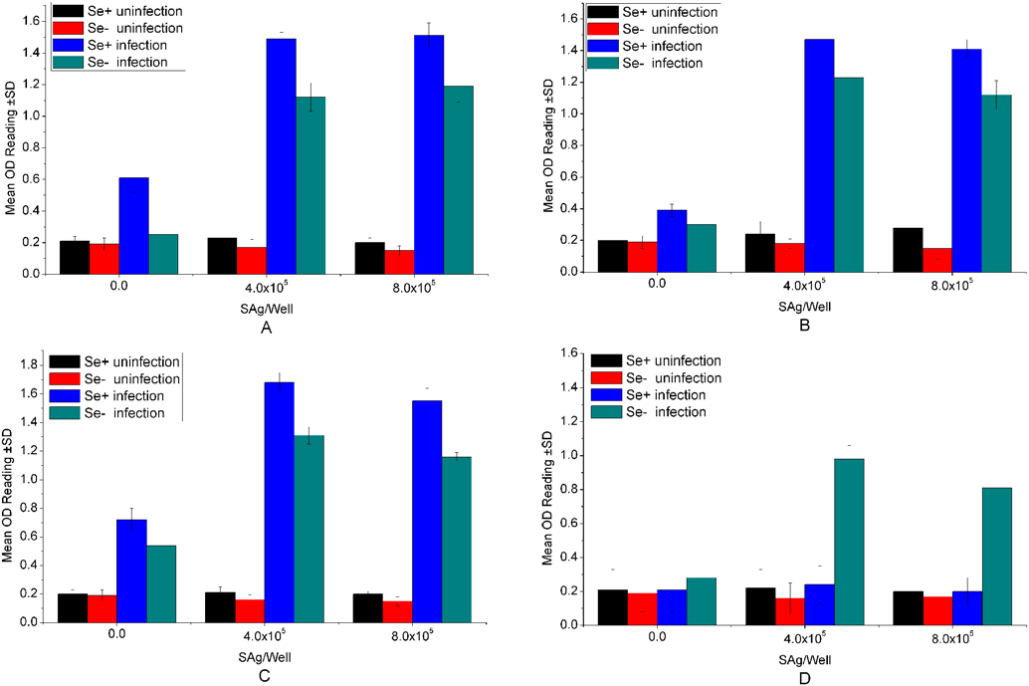
In vitro proliferation of MLN cells isolated from *Cryptosporidium parvum*-infected and uninfected (C57BL/6) mice following re-stimulation with SAg (Sporozoites Antigen). The MLN cells were isolated from uninfected and infected mice 7(A), 14(B), 21(C), and 28(D) days post-infection (DPI). Se^+^ and Se^−^ represent Se-adequate and –deficient mice, respectively. Results are expressed as mean OD of triplicate wells±standard deviation (SD).

To delineate whether a cell-mediated response develops during *C. parvum* infection in Se-deficient and Se-adequate mice, MLN cells were isolated at weekly intervals following infection and re-stimulated in vitro with *C. parvum* SAg (Sporozoites Antigen). The MLN cells isolated from infected Se-adequate mice 7, 14, or 21 days following infection markedly proliferated in response to *C. parvum* SAg (Figure 4 A, B and C). But proliferated in response to *C. parvum* SAg in Se-adequate mice at 28 days following infection was lower than Se-deficient mice at the dose of 4.0×10^5^ and 8.0×10^5^. *C. parvum*-specific proliferative immune responses were particularly detected at the infectivity phase of infection. The lower level of cellular immune responses at 4 weeks of infection in Se-adequate mice was correlated with the elimination of parasites from the gastrointestinal tract. Similarly, no difference in the proliferative response to SAg was observed in MLN cells isolated from Se-deficient mice 7, 14, 21 and 28 days among uninfected Se-adequate and –deficient mice (Figure 4). This confirms that the absence of Se has no effect in the generation of T-cell proliferative responses under the conditions of uninfection. Significant difference in the proliferative responses to SAg was observed between Se-deficient and Se-adequate mice models at days 7, 14, 21 or 28 days of infection. No proliferation of MLN cells isolated from infected or uninfected mice was observed following stimulation with the unrelated antigen, ovalbumin (data not shown). Also, no significant proliferation was observed in MLN cells isolated from control or Se-adequate mice following restimulation with SAg (data not shown).

### Cytokine production

**Figure 5:**
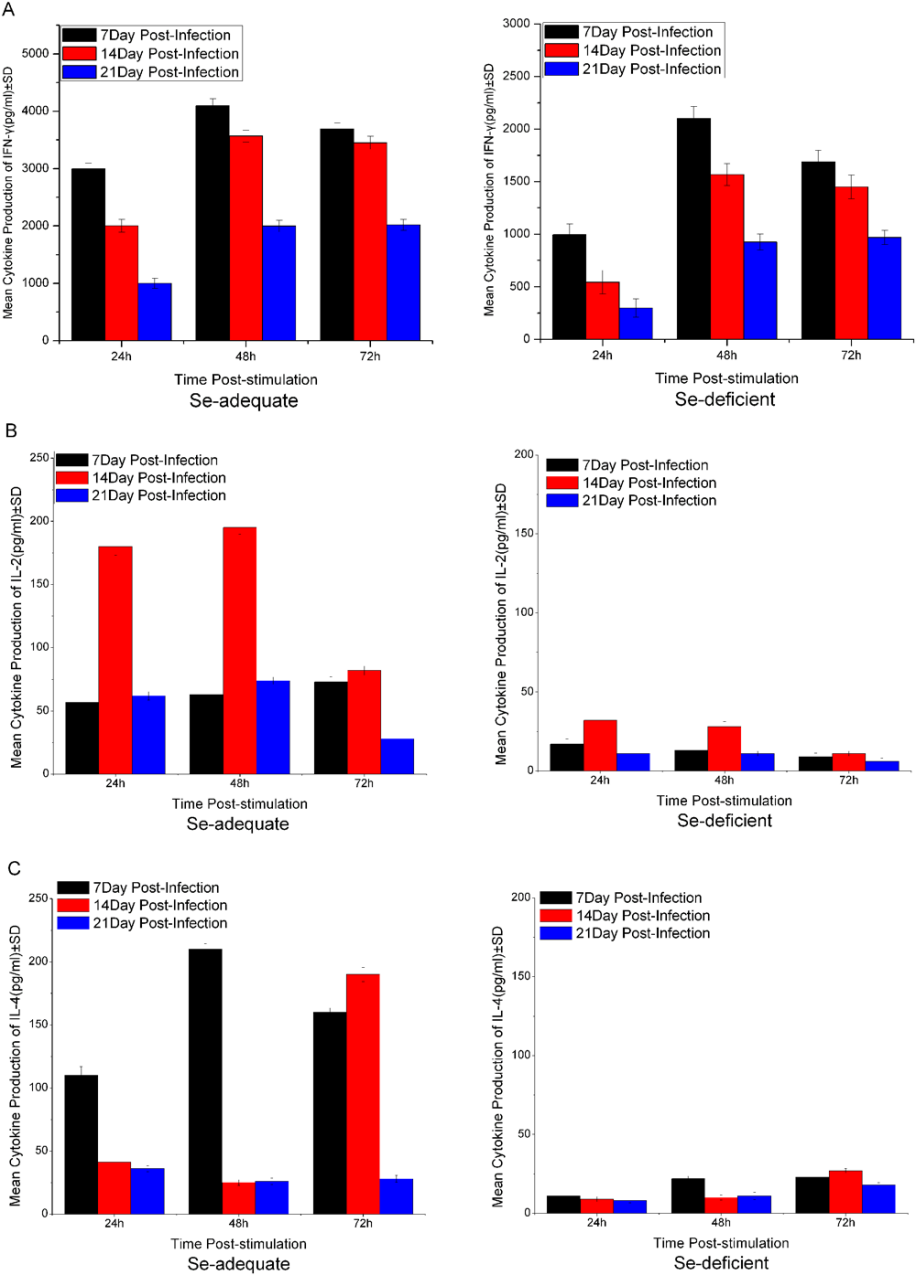
Kinetics of Th1 and Th2 cytokine profile following the infection of *C. parvum*. Th1 (IFN-γ(A), IL-2(B) and Th2 (IL-4) (C) cytokine profile of MLN cells isolated from *Cryptosporidium parvum*-infected Se-deficient and Se-adequate mice models following in vitro stimulation with SAg. The cytokine levels depicted were adjusted by subtracting the level of spontaneous cytokine release from SAg-stimulated MLN cells obtained from uninfected C57BL/6 mice. Results are expressed as mean production of cytokines±standard deviation (SD).

The nature of an acquired immune response to any infection is to a great extent determined by the balance between a Th1 and Th2 cytokine response. Cytokine secretion patterns of SAg-stimulated MLN cells from infected Se-deficient and Se-adequate mice were evaluated in terms of Th1 and/or Th2 response against *C. parvum*. Differential patterns of Th1 (IFN-γ (Figure 5A) and IL-2 (Figure 5B)) and Th2 (IL-4) (Figure 5C) Cytokines secretion was observed for MLN cells isolated from *C. parvum*-infected Se-deficient and Se-adequate mice.

The MLN cells from Se-adequate mice secreted significantly higher levels of IFN-γ (>1900 pg/ml) (Figure 5A) during the early period of infection (7 or 14 days) (P<0.01) and their levels decreased (500–1000 pg/ml) as the mice started recovering from the infection (21 days) (Figure 5A). Also, the concentration of IL-2 was significantly higher at the peak of oocyst shedding (14 days of infection) (P<0.01) than that at days 7 and 21 of infection in Se-adequate or –deficient mice. Whereas, MLN cells from Se-deficient mice expressed, as expected, lower level of IFN-γ and the expression level of IL-2 was markedly lower than those secreted by MLN cells from Se-adequate mice (P<0.01) at day 14 of infection, following 24–72 h of re-stimulation (Figure 5B).

Similarly, MLN cells from infected Se-adequate mice expressed higher secretion of IL-4 during the early period of infection (day 7 of infection) following 24–48 h of re-stimulation (P<0.01) than those isolated at days 14 or 21 of infection. In contrast, MLN cells from Se-deficient mice secreted significantly lower level of IL-4 (P<0.01) at day 7 of infection and the level did not increase further in those isolated at day 14 of infection (Figure 5C). Overall, the levels of Th1 cytokine (IFN-γ and IL-2) and Th2 cytokines (IL-4) in MLN cells isolated from Se-adequate mice were significantly higher than those from Se-deficient mice at the whole stage following *C. parvum* infection (Figure 5).

### Total leukocyte counts and CD3, CD4 and CD8 cell counts

**Figure 6:**
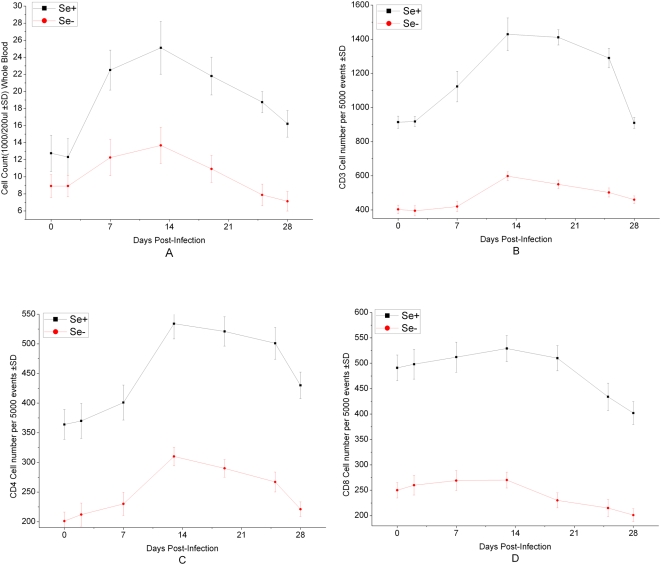
The Kinetic pattern of total leukocyte cell count (A), CD3 (B), CD4 (C) and CD8 (D) of Se adequate and Se deficient mice after *C. parvum* inoculation from day 0 to 28. There was significantly difference between two groups (p<0.01). Results were expressed as mean cell count±standard deviation (SD).

The total CD45 (common leukocyte antigen) white cell count increased gradually from the start of *C.parvum* inoculation to days 13, increasing from an average of 12,752 to 25,111 cells/200 µl in Se adequate group, a 96.9% of rise, while from an average of 8,913 to 13,672 cells/200 µl, a 53.4% of rise in Se deficient group. The total count then began to gradually fall from days 13 to days 28 in both Se adequate group and Se deficient group, the rate of fall is 35.5% and 47.9%, respectively (Figure 6A).

Before *C.parvum* inoculation (day 0), the CD3, CD4 and CD8 values in Se deficient group mice were decreased by 55.9%, 44.8% and 49.1%, respectively (Figure 6B,C,and D). After *C.parvum* inoculation, the dynamic patterns of CD3, CD4 and CD8 counts kept a rise before day 13 and a drop after day 13. The CD3 counts of day 0 kept approximately similar to the cell numbers of day 28, whereas the CD4 counts of day 0 were lower and the CD8 counts of day 0 were higher (Figure 6D). At day 27 and 28 *C. parvum* oocysts in all mice stools in Se adequate group were not observed, while at this time point *C. parvum* oocysts shedding remained higher level in Se deficient group. Generally, the counts of CD3, CD4 and CD8 in Se deficient group mice were significantly lower (p<0.01) during the whole experiments.

## Discussion

In some regions with lower soil selenium contents, the increasing number of some diseases in humans was observed in association with selenium deficiency [12], [13]. The selenium deficiency may affect the prevalence of parasitological diseases (such as *C.parvum*) and decrease efficiency of the immune system. Se can be carried to the fetus via the placenta [14] and to the newborn via the breast milk [15].

In this study Se deficiency mice model was performed by feeding 14 days pregnant female mice with Se deficient chow. GPx activity was measured in plasma samples as standard in valuating the Se status [16]. Mothers with feed of low Se chow and offspring are considered to be the status of Se deficiency by measurements of Se levels and of GPx activity (Figure 1).These differences of Se levels and GPx activities between the two groups proved that the method of Se depletion was feasible for experimental animals.

Comparative analysis was conducted between the Se-deficient and Se-adequate mice following the infection of *C.parvum*. Overall results showed that the activity of antioxidant (MDA, SOD, GSH and CAT) in Se-adequate mice was significantly higher than that of Se-deficient mice following the infection of *C.parvum*. It is suggested that the relativity might exist between Se deficiency and infection of parasites. Ribeiro et al. [17] suggested a decrease in GSH levels in host might render them susceptible to *schistosomula*. It may explain the susceptibility of host to infection of *C.parvum* due to a decrease in GSH levels of Se deficient mice in this study. The roles of selenoenzymes, GPx, in the intracellular and extracellular antioxidant defense systems have been well characterized. GPx isoenzymes use GSH as a donor of reducing equivalents to detoxify H_2_O_2_, peroxi-derivatives of various organic compounds (e.g., fatty acids, cholesterol, and phospholipids) [18], and peroxynitrite [19]. Thus, GPx is essential for maintenance of the cellular redox status and for the regulation of the redox-sensitive signaling and transcription mechanisms. Notably, although Se deficiency has been shown to suppress GPx expression and decrease the corresponding enzymatic activities, similar phenomenon was also observed from this study. Selenium deficiency could lead to enhanced lipid peroxidation through loss of selenium-dependent glutathione peroxidase activity [20].

The level of infection achieved in Se-deficient and Se-adequate mice models was sufficient to generate a parasite-specific immune response as evidenced by the parasite-specific proliferative responses obtained following ex vivo re-stimulation of MLN cells. In the Se-adequate mice model, the level of immune responses achieved was sufficient to clear their infection. In contrast, these weak proliferative responses might correlate with an unability to control this infection in Se-deficient mice. Thus, *C. parvum*-infected Se-deficient mice demonstrated heavy oocyst shedding, rapid weight loss, and dehydration within 28 days. Smith et al. [21] also showed similar in vitro proliferative responses using spleen cells isolated from infected GKO mice. Proliferative responses have also been detected in splenic lymphocytes from infected mice following multiple oral priming in vivo with *C. parvum* oocysts [22]. Host depletion of Se was associated to a reduced immune response [23], and an active immune response was essential to overcome the chronic phase of *C.parvum* infection.

An immunophenotypic analysis of the T-cell populations in the MLN cells responding to SAg has revealed that the major T-cell subsets activated in vitro are of CD4^+^ αβ^+^ TCR phenotype, followed by αβ^+^ TCR-carrying CD8^+^ T cells, in both mouse models (data not shown). B cells, which recognized only conformational epitopes, did not proliferate in response to SAg. The results in this study were similarity with Mead and You [24], where they reported increase in the percentage of CD4^+^ and CD8^+^ T-cell populations in spleens of infected GKO mice. Increased susceptibility to *C. parvum* infection of mice lacking specific αβ^+^ T-cell subsets [25] or MHC-II molecules [26] or immunocompetent mice treated with anti-CD4 antibodies [27], [28], [29] suggested that CD4^+^ T cells were more essential for the control of infection.

The level of Th1 and Th2 cytokines achieved by proliferated T cells revealed different patterns of expression in infected Se-adequate mice versus infected Se-deficient mice. In Se-adequate mice, the immune response appeared to be dominated by both Th1 and Th2 cytokines, as evident by increased expression of IFN-γ, IL-2, and IL-4 during the early stages of infection. This increase in IFN-γ expression remained high until recovery from the infection in Se-adequate mice. In consistence with the results in this study, IFN-γ was reported to appear earlier in the intestinal mucosa of mice infected with *C. parvum* [30] and has been shown to provide innate resistance to *C. parvum* infection [28], [31], [32], [33], [34], [35]. Although increased secretion of IL-2 was observed at the peak of oocysts shedding (day 14), the role of this cytokine in the termination of infection is unclear up to date. Enriquez and Sterling [36] reported that IL-2 plays an important role on decreasing in the level of oocyst excretion. So the longer time and the more number of oocysts in Se-deficient mice might correlate with lower level of IL-2. It is thus possible that Se deficiency could exacerbate the impairment of responses in infected mice fed with Se-deficient chow. Beck et al. [37] showed that lymphocyte proliferation to mitogen or antigens decreased in Se-deficient mice infected with a cardio virulent virus strain. Likewise, it is possible that Se-deficient mice could present low activity of T CD8^+^cells, pivotal cells for the protective response against *C.parvum* infection. Selenium was also shown to be important for the expression of high affinity IL-2 receptors in T cells [38], a mechanism that can also concur to the higher susceptibility presented by Se-deficient mice, since down regulation of IL2-R was verified in *C.parvum* infection in mice.

Early expression of IL-4 along with IFN-γ observed in Se-adequate mice has also been shown to be upregulated following infection of *C. parvum*. In *C. parvum* infection, IL-4 may regulate the secretion of IFN-γ, and hence sustain intestinal homeostasis by limiting the recruitment of inflammatory cells in response to infection. It has been shown in vitro that IL-4 can act synergistically with IFN-γ to inhibit the reproduction of *C. parvum* in an enterocyte cell line [39]. Increased susceptibility to *C. parvum* infection in mice after treatment with anti-IL-4 antibodies, or in mice lacking either an IL-4 gene or the gene for the IL-4 receptor [36], [40], supports the role of IL-4 cytokine in the control of infection. So lower level of IL-4 in Se-deficient mice might help increase susceptibility to *C. parvum* infection.

However, the expression of IL-2 and IL-4 was significantly lower than that observed in infected Se-deficient mice. It is likely that the decreased expression of Th2 cytokines in Se-deficient mice may have contributed to the *C. parvum*-induced severe enteritis within 2–3 weeks [21], [24], [30], [41], [42]. The results suggested that the expression of both Th1 and Th2 cytokines, as expressed in Se-adequate mice, were important in the resolution of *C. parvum* infection.

Trace elements modulate immune responses through their critical role in the enzyme activity. Both deficiency and excess of trace elements have been recognized as influencing immunity [43]. Recently, clinical applications concerning selenium show successful treatment of dermatosis [44] and intracellular parasites [45] after selenium supplementation. This study has also showed that Se-deficient mice could easily infect with *C.parvum*. The correlation between selenium concentration, immunity status and *C.parvum* infection has been observed by dynamic changes of CD3, CD4 and CD8 cells. Other research has showed selenium deficiency was demonstrated to impair the ability of neutrophils to kill *C. albicans* in vitro test [46]. There were also many reports on selenium deficiency and impaired leukocyte and lymphocyte responses in vitro although their relevance to a disease in vitro is unproven [47]. Several experimental studies suggest that severe selenium deficiency compromises T-cell dependent immune functions as blastogenic response to mitogens, decreases the antibody response [48].

Selenium has been linked to viral infections, enhanced T-cell functions and TNF-β induced increase in natural killer cell activity [49]. Most experimental studies on selenium-deficient animals report normal phagocytosis and altered bacterial capacity of neutrophils. The decrease in glutathione peroxidase activity of polymorphonuclear cells following selenium deficiency could explain some of these alterations [48]. Adequate dietary levels of the micronutrient Se are necessary for survival, whereas lower or higher levels of Se can be harmful to animal [50]. Larvae fed Se in the penultimate and ultimate instars were more resistant to viral infection than larvae not fed Se in the final instars [51]. Increased oxidative stress in the host can increase the virulence of the coxsackievirus. Dietary deficiency of either vitamin E or selenium increases the heart muscle damage caused by a myocarditic strain of coxsackievirus. Dietary deficiency of either vitamin E or selenium allows a benign amyocarditic strain of coxsackievirus to convert to a virulent myocarditic strain [52].

### Conclusions

The studies presented here demonstrate the kinetics and comparative analysis of *C. parvum*-specific cell-mediated immune responses to *C. parvum* SAg in Se-deficient and Se-adequate mouse models. It is worth noticing that that a nutritional Se deficiency can lead to a higher susceptibility of host to *C.parvum* infection and decrease immune responses, in other word induced susceptibility of host is associated with an impaired antioxidant system following infection with *C.parvum* in C57BL/6 Selenium deficient mice. However, further studies will be needed to understand the mechanism in effect of Se deficiency on infection of *C.parvum*.

## Materials and Methods

### Parasite and preparation of Sporozoites antigens

The *C.parvum* (Henan isolate) in this study was originally from Dr. Longxuan Zhang (Henan Agricultural University, Department of Animal Science, Zhengzhou city, Henan province) and maintained by passage through calves. To propagate the oocysts, the isolate was further passaged in DEX immunosuppressed mice. Oocysts were purified using discontinuous sucrose and cesium chloride gradients, then stored at 4°C until used.

The SAg (Sporozoites antigens) was prepared for the in vitro proliferation and cytokine assays as described previously [41]. Briefly, oocysts were excysted via incubation at 37°C for 1 h in Leibovitz L-15 medium (GIBCO BRL, Grand Island, NY, USA) containing 0.75% taurocolic acid (Sigma). The excysted sporozoites were separated from the unexcysted oocysts and empty shells by passage through a sterile 2.0-µm-pore size polycarbonate filter (Costar, Cambridge, MA). The purified sporozoites were washed twice with sterile PBS. The final pellet was resuspended in RPMI media, and the sporozoites were counted with a hemocytometer. Finally, SAg was prepared by repeated freeze-thaw cycles.

### Mouse models of selenium depletion

Se depletion was conducted according to Moreno-Reyes and Andrea P. de Souza protocol [53], [54]. All animal studies complied with the Guidelines for Ethical Conduct in the Care and Use of Experimental Animals, People's Republic of China and with the institution's policy. Briefly, mice were depleted in Se since the embryonic development, by feeding female C57BL/6 mice during pregnancy and lactation periods with chows containing different levels of Se. Both chows have all the recommended nutrients, such as vitamins, amino acids (52.5 g/kg), oligoelements and fatty acids (41.2 g/kg), differing only in the Se levels: 0.2 mg Se/kg (control chow with adequate Se level), and 0.005 mg Se/kg (Se-deficient chow). After weaning (21 days after birth), groups Control Mothers (n = 20) and groups Se deficient Mother (n = 20) were sacrificed and the plasma and organs collected. The female offspring (20 Se-adequate mice and 20 Se-deficient mice) were separated throughout the experiment.

### Inoculation of mice with *C. parvum oocysts*

The inoculums were free of bacteria. Twenty 8–10 weeks old offspring female mice were then orally inoculated with a single dose of approximately 10^5^ *C.parvum* oocysts in 0.2 ml of 0.15 M phosphate-buffered saline (PBS, pH 7.2) by using a 19-gauge gavage needle. Inoculated mice were monitored for oocyst shedding in fecal pellets per day. Fecal pellets were collected and then resuspended in a volume of 2.5% potassium dichromate approximately equal to twice that of the feces and stored at 4°C. Fecal suspensions were smeared onto microscope slides and examined microscopically in a blind fashion and count the number of oocysts numbers.

### Blood collection and blood cell FACS analysis

Blood preparation for flow cytometry analysis, necropsies and blood samples collection at different time point (day 0, 2, 7, 13, 18, 25, and 28) during infection were performed as previously described [55]. In order to get a concentrated sample of white blood cells, six heparin treated micro-hematocrit capillary tubes were each filled to 90% of their capacity (approximately 60 µl) with whole blood from each mouse and centrifuged in a micro-hematocrit centrifuge for 4 min. The six tubes were separated into two groups of three tubes each, so both sets of three tubes were approximately equal in total volume (180 µl). The total counts of white cell counts and CD4, CD8, CD3 cell counts were performed were analyzed as previously described [55]. Briefly, blood cells prepared from individual mice were stained with fluorescein isothiocyanate (FITC)-conjugated F(ab′)2 fragments of anti-Thy-1.2, anti-CD4, and anti-CD8 and anti-CD3 or were double-stained with FITC-conjugated anti-mouse Ig and phycoerythrin-conjugated anti-CD45R and analyzed by fluorescence activated cell sorter analysis (FACS) on a BD LSRII (BD Biosciences).FITC-conjugated F(ab′)2 fragments of anti-mouse Ig and phycoerythrin- conjugated anti-CD45R were purchased from Calbiochem (Merck). F(ab′)2 fragments of FITC-conjugated goat anti-rabbit Ig (Calbiochem, Merck) as a secondary antibody.

### Determination of selenium levels

Approximately 30 µl blood samples were collected in heparinized microcapillaries from the tail of each mouse at different time point (day 0, 2, 7, 13, 18, 25, and 28) post infection. Plasma samples were obtained after centrifugation of blood in a micro- hematocrit centrifuge and were stored at −20°C until analysis. The plasma levels of Se were measured by in Zeeman corrected atomic absorption spectrometry with a limit of sensitivity of 64 nM (5 ng/mL) according to method [54]. Undetectable concentrations were assigned a value of 5 ng/mL [53].

### Determination of antioxidant enzyme activity

The plasma samples for analysis of antioxidant enzyme activities were performed according to protocols reported previously. Briefly, GPx activity was measured by a coupled assay with yeast GR, using H_2_O_2_ as a substrate [56].CAT activity was determined by the method of Aebi that monitors the rate of H_2_O_2_ decomposition [57]. The total SOD activity was determined by the nitroblue tetrazolium assay of Spitz and Oberley [58]. malondialdehyde (MDA) concentration was measured by by spectrophotometry with thiobarbituric acid [59].

### Mesenteric lymph node cells

The MLN cells were harvested from *C. parvum*-infected and control C57BL/6 mice at days 7, 14, 21, and 28 of infection. These time points were selected to allow for a kinetic analysis of the immune response in Se-adequate mice and Se-deficient murine models. Single cell suspensions were prepared by passage of the MLN cells through a sterile wire mesh. The MLN cells from each group of mice were pooled together and finally suspended in complete RPMI 1640 (Sigma) containing 10% fetal calf serum, 200 mM L-glutamine, 100 U penicillin/streptomycin, and 5×10^−5^ M 2-mercaptoethanol.

### Proliferative assay

Proliferative responses of MLN cells from *C. parvum*-infected and control mice in response to SAg were measured. The MLN cells were plated at a concentration of 8×10^5^ cells per well in 96-well, flat-bottomed microtiter plates (Costar, Cambridge, MA, USA) and restimulated in vitro with two different concentrations of *C. parvum* SAg (antigen equivalent to 8×10^5^ and 4×10^5^ sporozoites) in a total volume of 200 µl of RPMI/well. To assess the antigenic specificity of the proliferative response, wells containing ovalbumin (100 µg/ml) were included. The plates were incubated in a humidified 5% CO_2_ atmosphere at 37°C for 5 days. Proliferation was determined by using a colorimetric 5-bromo-2′-deoxyuridine (BrdU) cell proliferation enzyme-linked immunosorbent assay (ELISA), (Roche Molecular Biochemicals, Mannheim, USA). This ELISA measures proliferation as a function of BrdU incorporation during DNA synthesis. The assay was performed according to the manufacturer's instructions. Briefly, 20 µl of BrdU labeling solution was added to each well for 3 h. The microtiter plates were then centrifuged (300 g, 15°C, 10 min). The supernatant was removed and the pelleted MLN cells were dried with a hair dryer. The cells were fixed and their DNA was denatured with a solution (provided in the kit) for 30 min. One hundred microliters of anti-BrdU peroxidase-conjugated antibody was added to each well and incubated for 90 min at room temperature. Wells were washed three times with PBS. Bound peroxidase was detected by addition of tetramethylbenzidine (TMB) substrate, provided in the kit. Thirty minutes following the addition of substrate, the reaction was stopped by adding 1 M H_2_SO_4_ and quantified by measuring the optical density OD_450_ nm using an ELISA reader (Molecular Devices, Sunnyvale, CA, USA).

### MLN cells cytokine analysis

Parallel cultures of MLN single cell suspensions were set up from *C. parvum*-infected mice along with their respective controls. The MLN cells were plated at a concentration of 8×10^5^ cells per well and re-stimulated in vitro with 4×10^5^ SAg in a total volume of 100 µl of RPMI/well. The MLN cells and the culture supernatant fluid were harvested at 24, 48, and 72 h after re-stimulation. The cell-free supernatant was stored at −80°C until used for cytokine analysis.

Th1 (IL-2 and IFN-γ) and Th2 (IL-4) cytokine concentrations were measured using the OptEIA ELISA kit (Merck). Briefly, flexible 96-well plates (Costar, Cambridge, MA, USA) were coated overnight at 4°C with the appropriate capture antibody diluted in 0.1 M NaHCO_3_ buffer, pH 9.6, according to the manufacturer's instructions. All remaining active sites were blocked with assay diluent for 60 min at room temperature. One hundred microliters of culture supernatant and cytokine standards were incubated for 120 min at room temperature, washed, and the bound cytokines were detected with the appropriate biotinylated anti-mouse antibody for different cytokines. The antibody was detected with avidin-horseradish peroxidase conjugate with TMB as the substrate (Calbiochem, Merck). The reaction was stopped with 2 N H_2_SO_4_ after 30 min and read at 450 nm using an ELISA reader (Molecular Devices, Sunnyvale, CA, USA). Cytokine secretion levels following SAg stimulation of MLN cells isolated from infected mice were adjusted by subtracting the background levels obtained following SAg stimulation of MLN cells isolated from control mice.

### Statistical analysis

All values are expressed as mean±standard deviations (SD). The cytokine concentrations at different time points and differences between Se-deficient and Se-adequate mice were analyzed by Student's t-test (unpaired). P<0.01 were considered statistically significant.
